# The Hamas massacre of Oct 7, 2023, and its aftermath, medical crimes, and *the Lancet* commission report on medicine, Nazism, and the Holocaust

**DOI:** 10.1186/s13584-024-00608-w

**Published:** 2024-04-12

**Authors:** Shmuel P. Reis, Hedy S. Wald

**Affiliations:** 1 Digital Medical Technologies, Holon Institute of Technology, 52 Golomb St, POB 305, Holon, 5810201 Israel; 2 Clinical Professor of Family Medicine, Warren Alpert Medical School of Brown University, Providence, RI USA

**Keywords:** Bioethics, The Moral education of the health professional, Medicine, Nazism and the Holocaust, Hamas October 7 massacre, 2023-4 War in Gaza, Crimes against humanity, Medical crimes

## Abstract

**Background:**

The report of the *Lancet* Commission on medicine, Nazism, and the Holocaust, released in November 2023, calls for this history to be required for all health professions education, to foster morally courageous health professionals who speak up when necessary.

**Main body:**

The report was released a month after Hamas’ October 7 invasion of Israel, with the accompanying massacre of over 1200 people, taking of civilian hostages, and gender-based violence. These acts constitute crimes against humanity including genocide. Post-October 7, war in Gaza resulted, with a legitimate objective of Israel defending itself within international law. The authors discuss an accompanying Statement to the report condemning Hamas crimes and denouncing the perpetrators’ use of their own civilians as human shields, including in healthcare facilities, and with the Hamas attack unleashing immense and ongoing suffering in Israel and beyond. With some exceptions, the medical literature shows a marked absence of condemnation of Hamas atrocities and includes unsubstantiated criticisms of Israel’s military. A significant surge in global antisemitism including on university campuses since October 7, 2023, has occurred; and health professionals, according to the Commission, have a special responsibility to fight antisemitism and discrimination of all kinds. In this context, the authors discuss the controversy and criticism regarding diversity, equity, and inclusion education programs (“DEI”) including such programs failing to protect Jews on campuses, especially as the U.S. President Biden’s “The U.S. National Strategy to Counter Antisemitism,” released in May 2023, calls for the inclusion of issues of antisemitism and religious discrimination within all DEI education programs. The authors support an evidence-based approach to the Hamas massacre, its aftermath and its relevance to health professionals both within medicine and their global citizenship, including refuting the international community accusations and anti-Israel libel.

**Conclusions:**

The report of *the Lancet* Commission on medicine, Nazism, and the Holocaust has striking relevance to the Hamas massacre of October 7, 2023 and its aftermath. This is further conveyed in an accompanying Statement, that describes the report’s implications for contemporary medicine, including: 1) provision of skills required to detect and prevent crimes against humanity and genocide; (2) care for victims of atrocities; (3) upholding the healing ethos central to the practice of medicine; and (4) fostering history-informed morally courageous health professionals who speak up when necessary.

## Background

The recently released report of *The Lancet* Commission on medicine, Nazism, and the Holocaust [1] calls for the teaching of this tragic and distinctive history to be required in all health professions education in order to foster health professionals who are ethically resilient, resistant to coercion and temptation, and morally courageous, speaking up when necessary [1]. It thus addresses the moral education of health professionals, including underlining their unique responsibilities as well as those of their academic institutions and professional associations to eliminate antisemitism, racism, and all forms of discrimination [1]. The inclusion of medicine and the Holocaust in the curriculum is meant to “….provide an opportunity to reflect on the role of the doctor in society” [2]. In this vein, the report also recommends adding the detection and prevention of war crimes, mass atrocities, human rights violations, crimes against humanity and genocide to the ethos of medicine and desired health professionals’ competencies [1, 3].

## Main text

The report was released on November 8, 2023, shortly after Hamas’s October 7, 2023, invasion of Israel with massacre of over 1200 people, mostly civilians, and the kidnapping of civilian hostages (including babies, children, women, and older persons, many with complex medical conditions). No International Committee of Red Cross (ICRC) visits the hostages and limited medical care, food, and water as well as inhumane incarceration conditions and physical and psychological violence, compound the abduction crime. Furthermore, over a hundred thousand Israelis have been displaced from the country’s southern and northern borders with resulting disruption of employment, education, and healthcare. This massacre included clear and flagrant crimes against humanity and furthermore, as described in a letter to the former president of Harvard University from the leadership of the Hebrew University of Jerusalem, “the Hamas leaders’ explicit statements, as well as their actions, provide a clear indication that the mass killing was committed with the intent to destroy the Jews in Israel. One does not have to be an international law expert to realize extreme immorality of this crime of genocide” [4]. In addition, Hamas deliberately puts Palestinian civilians in harm’s way creating significant challenges for the Israel Defense Forces which seeks to minimize civilian casualties and unlike Hamas, distinguishes between civilians and combatants” [5].

*The Lancet* Commission report and its universal messages have striking global and local relevance with timely implications and recommendations. It hopes to engender a “critical reflection on the connections between the historical and contemporary values and ethics of health professionals” [1] as part of professional training which impacts health professionals’ role in society including global conflicts. Grappling with the history of the medical profession’s pervasive complicity in Nazism and the Holocaust supports development of morally conscious and resilient health professionals who will thus be able to dispel false healthcare narratives with factual misinformation [6] and who would subsequently act as evidence-based, [6] morally grounded “enlightened change agents” [7].

### Background: October 7 and war in Gaza

Immediately following the events of October 7, a war in Gaza resulted with a legitimate objective of defense by Israel (for the country’s very existence) within international law [6]. This challenge included the targeted destruction of a vast, highly developed, and dangerous tunnel network built with humanitarian aid [8]. Furthermore, the widespread weaponization of Gaza humanitarian infrastructure was revealed including hospitals, health clinics, houses of worship, educational institutions, and the private apartments of UNRWA teachers and physicians [6, 8]. More specifically, hospitals have been and are used intentionally by Hamas as command centers, weapons and transport depots, hostage prisons, launching sites for rockets and as bases from which terrorist attacks including the October 7 massacre were launched and to which the perpetrators returned with hostages and loot [5, 6].

As a hospital director recruited to Hamas confessed, “there are employees [of our hospital] who are military operatives of the Izz ad-Din al-Qassam Brigades [the armed wing of the Hamas terrorist organization] – doctors, nurses, paramedics, clerks, and staff members” [9]. According to the rules of war, healthcare facilities are meant to be protected spaces, even during combat. It is, however, a war crime when one is used in any way to support hostile activity or for fighters to hide among patients. According to the Geneva Conventions, in such circumstances a hospital will lose its immunity [10]. Within these difficult circumstances, Israel has called for evacuation of hospitals before any military action [11] was taken against enemy combatants hiding in these healthcare facilities.

The laws of war are further challenged by the widespread Hamas practice of using non-combatants as human shields. There are many such examples, all well documented, including encouraging tens of thousands to find shelter in hospitals (despite the terrorist infrastructure located within the premises and underground), forcibly preventing civilians from leaving Northern Gaza thus interfering with Israel Defense Forces’ evacuation instructions meant to help civilians avoiding harm, and including women and children in lookout posts prior to terrorist attacks [6]. Thousands of long- and short-range missiles and mortars continue to be fired indiscriminately by Hamas from Gaza into Israeli civilian areas. Each launch a war crime on its own [12]. Hamas has also committed multiple medical crimes. These include killing Israeli physicians, nurses and paramedics tending to casualties and disabling Israeli ambulances purposefully on October 7, free use of Gaza ambulances to transport terrorists, weapons, as well as hostages against their will, and widespread collusion of health professionals with Hamas terrorists. Evidence exists of holding hostages on hospital premises and at least one hostage murdered there [9–11, 13]. The most significant crime committed by Hamas before, during, and after October 7 is the cruel manipulation of the core values and morality of civilization within and beyond codes and laws. Some examples include (1) placing non-combatants including women and children in harm’s way, negating military ethos to abide by humanitarian principles, in order to fuel negative anti-Israel propaganda [6], (2) psychological terrorism enacted via the broadcast of selective footage of taking of hostages, omitting the origin and nature of the criminal abductions, the unlawful imprisonment and inhumane conditions, the use of hostages as human shields as well as their murder [12], (3) fuel, food, water and medical supplies that are meant for Gazan civilians being tragically diverted at gunpoint by Hamas for their own benefit under the guise of humanitarian and healthcare needs [5], (4) toys (e.g. teddy bears) either booby-trapped or used as hiding places for weapons, (5) children’s bedrooms and hospital neonatal intensive care units (NICUs) serving as weapon and explosives depots [13], and (6) evidence of Hamas possession of hundreds of millions of shekels and dollars in cash in safes within apartments and tunnels in service of the terror infrastructure while adamantly placing the responsibility for humanitarian supplies on the rest of the world including Israel [14].

The impact of such weaponization of values, travesty of ethics, and manipulation of the empathy, compassion, and morality of many for terrorist purposes is far-reaching with dire consequences including contributing to atrocities, civilian suffering, and significantly increased antisemitism. Evidence of war crimes by Hamas abounds and perpetrators must be held accountable [5, 6, 8, 9, 10, 11, 13, 15].

Concern for the suffering of civilians on both sides of the Israel-Gaza border is truly agonizing with ongoing issues of trauma amongst Israelis due to the massacre and hostage-taking, vast numbers of displaced Israelis from the south and north, and a humanitarian crisis in Gaza. While “Hamas’ clear culpability for events in and since October 7, 2023,” is noted [16] including dehumanization of Jews and their own civilians, concerns persist from the international community about Israel’s actions in relation to the Gaza humanitarian crisis, conduct of war within international law, and raids of healthcare facilities. “Vilifying Israel” within this is “obfuscation of the truth” [16]. Evidence indicates that (1) a significant amount of allowed humanitarian aid to Gaza is diverted to Hamas for their terrorist infrastructure; [17] (2) On October 7, Hamas unilaterally and barbarically breached a ceasefire that had generally held through October 6 [18]; (3) in this context, Israel has rejected international calls for ceasefire since they are not accompanied by surrender and hostage release by Hamas; [10] (4) all military actions (including those against healthcare facilities) are subject to legal counsel with only intelligence flagged targets hit, and Israel, in general, entitled to act in legitimate self-defense [19, 20].

### A perspective on implications of the report of the Lancet Commission on medicine, Nazism, and the Holocaust

History-informed professional identity formation, as introduced and elaborated on within *the Lancet* Commission report, states that the moral responsibility for speaking up is essential for the health professional [1]. The Statement accompanying the report thus condemns Hamas’ crimes “in the strongest terms,” and denounces the “perpetrators’ use of their own people (including patients and staff in health-care facilities) as human shields” [3]. Expressing compassion for Gazan non-combatants, the Statement further acknowledges how “this antisemitic attack by Hamas has unleashed immense and ongoing suffering in Israel and beyond and has led to a reality in Gaza in which healthcare workers’ lives and delivery of health care are threatened” [3].

Speaking up against these mass atrocities must include public condemnation of a well-documented widespread campaign of horrifying gender-based sexual violence against women; and some of the documentation comes from the terrorists themselves. This sadly continues to be a “widely disregarded issue” with “deafening silence of organizations tasked with safeguarding women and children from gender-based violence” [21]. Sexual violence war crimes continue in the abuse of hostages by Hamas terrorists [22].

Bringing documented evidence to respond to and correct erroneous stories in the media is within our professional remit. An instructive example is the ongoing libelous accusation that Israel purposely bombed the Al Ahli Arab Hospital causing hundreds of deaths. Shortly after this tragic event it was demonstrated to have been the result of a failed rocket launch by local terrorists. Not all those who falsely accuse Israel of this Palestinian terrorist crime have recanted [23].

Further examples of a false rhetoric accusing Israel of deliberately targeting hospital facilities without any regard for civilians must be replaced with the evidence that warnings were provided to evacuate due to the imperative to destroy tunnels beneath hospitals serving as terrorist command control centers and sites for Hamas terrorists seeking refuge [9–11, 13]. Further evidence includes (1) the major Gaza hospital (Al-Shifa) being ultimately evacuated of the estimated 50,000 civilians taking refuge there (most serving as human shields) as well as most of the patients (including 31 premature babies) [24] and staff without a single shot fired, and (2) collusion of the hospital personnel with terrorism including murder of a hostage on the hospital grounds [9–11, 13, 25].

Examples of omission as well as commission in published scholarship compromise our professional work. For example, a publication on health professionals and the war in the Middle East that appeared in a respected medical journal omitted descriptions of war crimes including violent kidnapping with lack of access to International Red Cross for hostages and “tens of thousands of Gazan missiles being fired from civilian locations including hospitals and schools now and for many years into Israeli cities and hospitals” [26, 27]. Health professionals and health professions’ trainees also have a professional responsibility to refrain from unprofessional and even inflammatory social media conduct including antisemitic postings.

*The Lancet* Commission Student Advisory Council elaborated further on the report’s relevance to the tragic events of October 7 in their publication “Why health-care learners and professionals should want to learn about medicine, Nazism, and the Holocaust” [28] Their publication, which accompanied the Commission report, stated that “the moral lessons of medicine, Nazism, and the Holocaust were called upon on October 7, 2023, the highest single-day loss of Jewish life since the Holocaust” [28]. As such, there is an urgent need to grapple with and learn from this history in order to properly equip our learners with a functioning moral compass [29]. Their article goes on to say, “Some reactions to this massacre, including in health-care facilities, academia, and the media, show that antisemitism is still prevalent in certain settings and poses threats to Jewish people everywhere. Humanitarian acts are vital to uphold the rights of all civilians and should include the immediate, safe return of all Israeli hostages held in Gaza as it is a humanitarian act protecting Jewish people’s right for life. All share the responsibility to protect innocent lives, Israeli and Palestinian, at all costs” [28].

### Medical journals, academia, and the war in Gaza

Aside from *the Lancet*, there has been a striking absence of public condemnation of Hamas terrorist atrocities and their implications in the major medical journals. The silence has been deafening in contrast to coverage of other world events calling for speaking up when necessary. For example, both The New England Journal of Medicine and British Medical Journal, published exposés of Russia’s blatant disregard for human rights in Ukraine [30, 31]. Yet no parallel condemnation of the October 7 Hamas terrorist massacre as well as decades of indiscriminate rockets fired on Israel [15] is forthcoming in these and other respected publications. Despite lack of coverage of the results of the “shocking outbreak of hostilities which began on October 7 with the Hamas pogrom perpetrated mostly on civilians (including many older persons)” [32] and impact of the trauma on mental health and healthcare in general, an exception that proves the rule can be found in Clarfield’s documentation of the challenges involved in caring for the vulnerable older population in Israel during wartime [32]. The appearance of this article in the Journal of the American Geriatric Society is an encouraging sign of journal integrity [32]. In his words, “innocents on both sides of the border – young and old – are paying a terrible price for that choice [citizens of the Gaza Strip electing Hamas] and Hamas’ inhumane acts.” While patently false and inflammatory narratives persist, such as coverage of the Al Ahli Arab hospital incident as noted above [23], concerns for all innocent victims as well as coverage of longstanding care of Palestinians by Israeli physicians in “war and peace” [33] have been published in *the Lancet.* Omission of key facts, however, appears even in this journal [34, 35]. Striking omissions in *the Lancet*, for example, are noted in a recent correspondence from UNRWA personnel about their challenges providing healthcare in Gaza [36]. These include UNRWA employees having participated in the Oct 7 massacre (allegedly 12% were official Hamas members), and the Hamas server farm being located beneath UNRWA headquarters in Gaza with cable connection into the headquarters, thus rendering UNRWA an accomplice to terrorism [37]. A letter with such glaring omissions becomes an object of disinformation with anti-Israel libel [16]. Another example of denouncing such omission is a letter in response to an “offline” Lancet editorial which referenced World Health Organization (WHO) chair’s “moral clarity” [35] within lack of key additional contextual facts [16].

Lancet editor-in-chief Richard Horton’s description of the urgent need for medicine and the Holocaust education as a “medical imperative” [2] in health professions education and subsequent establishment of the Lancet Commission on medicine, Nazism, and the Holocaust is spot-on. The Commission report recounts the history of the medical profession’s pervasive complicity in the Nazi atrocities including the Holocaust, underlining the unique responsibilities of health professionals, their academic institutions, and professional associations for eliminating antisemitism and all forms of discrimination. It therefore informs efforts to confront, and ideally eliminate, the alarming increase of antisemitism since the attacks of October 7 [38], notably on U.S. college campuses [39], and including medical campuses globally [40, 41] and in society in general.

In a recent open letter to university presidents, the Anti-Defamation League called for “enforcing codes of conduct in the wake of a flood of antisemitic harassment and discrimination (and hate) on campus since October 7” [38, 42]. In line with messages of the report, the health professions must be free of antisemitic hate.

In regard to recent moral failures within Congressional testimony by presidents of three top tier U.S. universities regarding campus antisemitism and calls for genocide against Jews (followed by the resignation of two of them), Weiner writes in the *Wall Street Journal* that the fact that “lawmakers had to ask about students’ genocidal sympathies at all reveals that some of our most prestigious universities are abjectly failing to cultivate virtue or wisdom” [42]. In calling out the “timidity of many university leaders in condemning the Hamas massacre and antisemitism more generally” [42], bioethicist Ezekiel Emmanuel advises: “we need to ask ourselves: What is in our curriculums? What do we think it means to be well educated?” [43] Furthermore, in line with the relevance of the Commission report, Ferguson reminds us that “anyone who has a naïve belief in the power of higher education to instill morality has not studied the history of German universities in the Third Reich” [44]. He elaborates how, “In Germany, to use the legalistic language of 2023, ‘speech crossed into conduct.’ The ‘final solution of the Jewish question’ began as speech - to be precise, it began as lectures and monographs and scholarly articles. It began in the songs of student fraternities. However with extraordinary speed after 1933, it crossed into conduct: first, systematic pseudo-legal discrimination and ultimately, a program of technocratic genocide” [45]. History informs us that even within Germany of the 1930s, so highly developed in academia with many Nobel laureates, the Nazification of medical education and the health professions [1] espoused ethics codes in medical faculties which included teaching about the unequal worth of human beings and the moral imperative of preserving a “pure” Aryan people. The Zeitgeist supported the authoritarian role of the physician, and the priority of a twisted public health over that of humane individual patient care [46]. Indeed, “teaching the Holocaust provides an opportunity for medicine to play its part in fighting antisemitism [2].

Contributing to many of these problems has been the unintended consequences of diversity, equity, and inclusion (DEI) campus programs which, while intending to solve one problem of discrimination, have led to others, including the exclusion of Jews. For this reason, there is growing concern about these programs, and in some cases frank opposition to them [47]. During the abovementioned U.S. Congressional hearings during which three U.S. university presidents testified about antisemitism on campuses, they were asked about the phenomenon of “DEI programs failing to protect Jewish communities” on U.S. campuses [47]. Debates on the viability of DEI programs “which seek to boost representation of historically underrepresented students and faculty at colleges, businesses and government agencies” [48] include judging such programs “irredeemable” due to faulty assumptions with a need for “dismantling DEI ideology” [49]. On the other hand, some call for an expansion of DEI programs to incorporate Jews and the fight against antisemitism into the DEI infrastructure [50]. Along these lines, President Biden’s recently published “U.S. National Strategy to Counter Antisemitism” calls for antisemitism and religious discrimination to be required within all DEI education programs [50]. Following the intense criticism of its DEI policy, Harvard University recently announced adding antisemitism to their DEI education [51]. Still, concerns persist about “the way in which some DEI efforts are undermining American constitutional values” [52] with some states in the U.S. recently passing legislation banning various DEI programs.

Within medical education per se, eliminating antisemitism and religious discrimination are notably absent, for example, from the “DEI competencies” of the American Association of Medical Colleges (AAMC) [52, 53]. Such competencies can and should be history-informed. In line with the recommendations of *the Lancet* Commission, we strongly recommend that that the tragic narrative of medicine during Nazism and the Holocaust be included in DEI education programs of all health professions education.

## Conclusions

*The Lancet* Commission report on medicine, Nazism, and the Holocaust has implications for today and teaching for tomorrow. It is relevant to understanding the ethical ramifications of the massacre of October 7 and its aftermath, including serving as a basis for fighting antisemitism and other forms of discrimination, especially within the current global significant surge. The Statement accompanying *the Lancet* Commission report [3] aptly captures the timely relevance of this report to the Hamas terrorist massacre of October 7, 2023, and its aftermath. The Statement emphasizes the key messages of *the Lancet* Commission Report, which are: “(1) to provide the skills required to detect and prevent crimes against humanity and genocide; (2) to care for the victims of atrocities; (3) to uphold the healing ethos central to the practice of medicine; and (4) to foster history-informed morally courageous health professionals who will speak up when necessary” [1]. The Statement continues: “All health care professionals and all of humanity need to come together in the hope of one day achieving a world free of terrorism, medical crimes, antisemitism, racism, and discrimination of any form” [3].

## Data Availability

Data sharing Not applicable.

## Abbreviations

DEI: Diversity, Equity, and Inclusion
ICRC: International Committee of Red Cross
UNRWA: The United Nations Relief and Works Agency for Palestine Refugees in the Near East
NICU: Neonatal Intensive Care Unit
U.S: United States
AAMC: American Association of Medical Colleges
