# Correction to: The Hamas massacre of Oct 7, 2023, and its aftermath, medical crimes, and the Lancet commission report on medicine, Nazism, and the Holocaust

**DOI:** 10.1186/s13584-024-00610-2

**Published:** 2024-05-07

**Authors:** Shmuel P. Reis, Hedy S. Wald

**Affiliations:** 1 Digital Medical Technologies, Holon Institute of Technology, 52 Golomb St, POB 305, Holon, 5810201 Israel; 2 Warren Alpert Medical School of Brown University, Providence, RI USA

**Correction to: Israel Journal of Health Policy Research (2024) 13:19**

10.1186/s13584-024-00608-w

The original publication of this article contained an incorrect disclaimer, the incorrect and correct information is listed in this correction article. The original article has been updated.

Incorrect

The views presented in this article do not necessarily represent the views of the editors or the reviewers of the journal.

Correct

The views presented in this article do not necessarily represent the views of the editors, the reviewers of the journal, or of the other members of the Lancet Commission on medicine, Nazism, and the Holocaust, but rather of the authors only

